# MoS_2_ nanobelts-carbon hybrid material for supercapacitor applications

**DOI:** 10.3389/fchem.2023.1166544

**Published:** 2023-08-22

**Authors:** Lina N. Khandare, Dattatray J. Late, Nandu B. Chaure

**Affiliations:** ^1^ Department of Physics, Savitribai Phule Pune University, Pune, India; ^2^ Centre for Nanoscience and Nanotechnology, Amity University Maharashtra, Mumbai, India

**Keywords:** lemon grass-derived carbon, MoS_2_, nanobelts, hybrid material, supercapacitor

## Abstract

The MoS_2_ nanobelts/Carbon hybrid nanostructure was synthesized by the simple hydrothermal method. The MoS_2_ nanobelts were distributed in the interlayers of Lemon grass-derived carbon (LG-C), provides the active sites and avoid restacking of the sheets. The structural and morphological characterization of MoS_2_/LG-C and LG-C were performed by Raman spectroscopy, X-ray diffraction, field emission scanning electron microscopy, transmission electron microscopy, and X-ray photoelectron spectroscopy. The electrochemical measurements were studied with cyclic voltammetry, the galvanostatic charge-discharge method, and electrochemical impedance spectroscopy. The specific capacitance of MoS_2_/LG-C and LG-C exhibits 77.5 F g^−1^ and 30.1 F g^−1^ at a current density of 0.5 A g^−1^. The MoS_2_/LG-C-based supercapacitor provided the maximum power density and energy density of 273.2 W kg^−1^ and 2.1 Wh kg^−1^, respectively. Furthermore, the cyclic stability of MoS_2_/LG-C was tested using charging-discharging up to 3,000 cycles, confirming only a 71.6% capacitance retention at a current density of 3 A g^−1^. The result showed that MoS_2_/LG-C is a superior low-cost electrode material that delivered a high electrochemical performance for the next generation of electrochemical energy storage.

## 1 Introduction

The development of energy storage devices, like rechargeable batteries, is important to produce environmentally friendly products (Huang et al., 2019; Shaqsi et al., 2020; Li et al., 2021). Nowadays, electrochemical energy storage devices such as supercapacitors and photovoltaic cells are commonly used (Kandasamy et al., 2021; Agrawal et al., 2022). Supercapacitors exhibit a quick charge storage property, which may contribute to their reduced charging times, greater cyclability, and therefore higher specific capacitance (Purkait et al., 2018; Narthana et al., 2021; Rajagopal et al., 2022; Rawat et al., 2022). The ongoing research effects focus on the production of a low-cost, long-life cycle, and high-specific capacitance material for the development of supercapacitors (Iro et al., 2016; Tomy et al., 2021). The selection of appropriate electrode material is important to improve the supercapacitor performance. Numerous materials have been used to store charges for electrochemical energy storage devices (Mohammed et al., 2019; Santoro et al., 2019). The store charges in supercapacitor devices are classified into three types, electrostatic double-layer capacitors, pseudocapacitors, and hybrid supercapacitors (Mathis et al., 2019; Atta and Fahim, 2021; Volfkovich, 2021). The double layer formed at the electrode surface is used for the storage of charges in electrostatic double-layer capacitors. In pseudocapacitors, charge storage occurs through a redox reaction (Fleischmann et al., 2022; Kour et al., 2022).

Biomass-derived carbon sources have natural renewable resources and are widely used because they are affordable, readily available, simple to prepare, and ecologically beneficial. Some examples of biomass that may be used as a resource for activated carbon for electrochemical energy storage include coconut oil, bamboo fiber, bean dregs, mango leaves, peanut shells, *etc.*, (Madhu et al., 2014; Ruan et al., 2014; Gaddam et al., 2016; Ji et al., 2018; Bai et al., 2020) In contrast, activated carbons frequently exhibit less capacitive characteristics, such as low conductivity and restricted charge flow rates. Activated carbon exhibits poor conductivity and limited charge storage capacity (Gomes Ferreira de Paula et al., 2019; Mohammed et al., 2019; Santoro et al., 2019; Luo et al., 2021). To overcome these difficulties, it is necessary to develop structural modifications as well as hybrid materials (Khandare and Late, 2017; Tomboc et al., 2020).

The Lemon Grass (Cymbopogon citratus) plant is known for its long leaves, which are specially used for making oil and spices. In Asian countries, Lemon Grass (LG) is used to provide taste and flavor to drinks (including tea, coffee, *etc.*). Among them, it has been utilized as a biofertilizer and feedstock. LG consists mostly of cellulose, which is considered to be a carbon source material with high potential (Liakos et al., 2016; Thota et al., 2018). The production of carbon and inorganic-based composites is a successful method for enhancing electrochemical energy storage by combining various inorganic materials, including transition metal oxides, and transition metal dichalcogenides, with carbon-based materials (Wang et al., 2020; Kour et al., 2022).

Molybdenum (Mo) has a variable oxidation state that can vary from +2 to +6. Molybdenum disulfide (MoS_2_) has a layered structure that shows pseudocapacitive behaviour to obtain high specific capacitance (Cook et al., 2017). Mo has been stacked in between two sulfur atoms (S-Mo-S) by weak van der Waals forces, which allow the electrolyte ions to intercalate in MoS_2_ (Kukkar et al., 2016). The ion diffusion works with MoS_2_ to store the charge in its faradic capacitive nature, which helps to improve the chance of storing the charge (Mahmood et al., 2016).

In this work, we report the low-cost synthesis of MoS_2_/Lemon Grass-derived carbon (MoS_2_/LG-C) hybrid material for enhancing the electrochemical performance. *In situ* growth of MoS_2_ nanobelts on surfaces of the LG-C through a facile redox reaction between ammonium molybdate and thiourea with carbon. The morphology and structural properties of LG-C and MoS_2_/LG-C hybrid materials are examined. The electrochemical measurements performed with cyclic voltammetry, galvanostatic charge-discharge testing, and cyclic stability to obtain the superior charge storage capacity in supercapacitors are discussed.

## 2 Experimental

### 2.1 Materials

Ammonium heptamolybdate tetrahydrate ((NH_4_)_6_Mo_7_O_24_.4H_2_O), thiourea (NH_2_CSNH_2_), and potassium hydroxide (KOH) are procured from HPCL, India. All chemicals were AR-grade (99% purity) and used without further purification. Overall, double-distilled (DD) water was used in the experiments.

### 2.2 Preparation of LG-C

The lemon grass (LG) leaves were collected from the local market based in Pune, India. Collected LG leaves were washed with DD water and dried in the sunlight for at least 2 days. The dried leaves were crushed finely and subsequently treated in KOH (1:1 weight ratio of LG leaf powder to KOH) for impregnation. After impregnation, the pH of the powder has been adjusted to 7 (neutral) by using a 1M HCl solution. After adjusting the pH, the LG powder was dried for 12 h at 80°C. Finally, the powder was kept in tubular furnaces in an argon atmosphere at 800°C for 3 h. The temperature of the tubular furnace was increased slowly by 5°C per minute and cooled naturally to room temperature (RT).

### 2.3 Preparation of MoS_2_/LG-C

The preparation was initiated by dissolving 100 mg of LG-C in 50 mL of DD water. The ammonium heptamolybdate tetrahydride and thiourea in quantities of 151 mg and 200 mg were added to the above reaction mixture, and the mixture was stirred at room temperature for 2 h. The obtained mixture was poured into the Teflon link autoclave and treated hydrothermally at 220°C for 24 h before being allowed to cool down naturally to RT. The suspension was centrifuged and washed in deionized water and ethanol. The final product was dried in the oven for 12 h at 80°C.

### 2.4 Material characterization

The Raman spectra were recorded at a 532 nm He-Ne laser source using a Renishaw InVia Raman microscope. The crystalline phase and crystal structure of the pristine and hybrid materials were examined using X-ray diffraction (XRD) on a Bruker D8 Advance X-ray diffractometer using Cu Kα (= 1.5405 Å) irradiation. FESEM images were captured using the FEI Nova NANOSEM 450. X-ray photoelectron spectroscopy (XPS) was recorded on the PHI Versaprobe III by using Al Ka X-rays. Transmission electron microscopy (TEM), high resolution (HRTEM), and energy dispersive X-ray spectroscopy (EDS) were recorded using the FEI Talos F200S instrument at 200 kV.

### 2.5 Electrode preparation

The electrodes were prepared on carbon paper as a collector. A 1 × 1 cm^2^ area of carbon paper was coated with a slurry of active electrode material, carbon acetylene, and polyvinylidene fluoride (PVDF) in ratio of 85:10:5, respectively. PVDF is used as a binder in dimethylformamide (DMF). The electrodes were dried in a hot air oven for 12 h at 120°C. The active material in the quantity of 1 mg was used for the supercapacitor electrode fabrication.

### 2.6 Electrochemical measurement

All electrochemical measurements were carried out at the Biologic SP-300 potentiostat/galvanostat using a three-electrode set-up and symmetric system in 1M Li_2_SO_4_ electrolyte. Ag/AgCl, platinum and LG-C, and MoS_2_/LG-C were used as reference, counter, and working electrodes, respectively. Cyclic voltammetry (CV), the galvanostatic charge-discharge (GCD) test, electrochemical impedance spectroscopy (EIS), and cyclic stability using the GCD test were examined to study the various supercapacitor parameters.

## 3 Results and discussion

**FIGURE 1:**
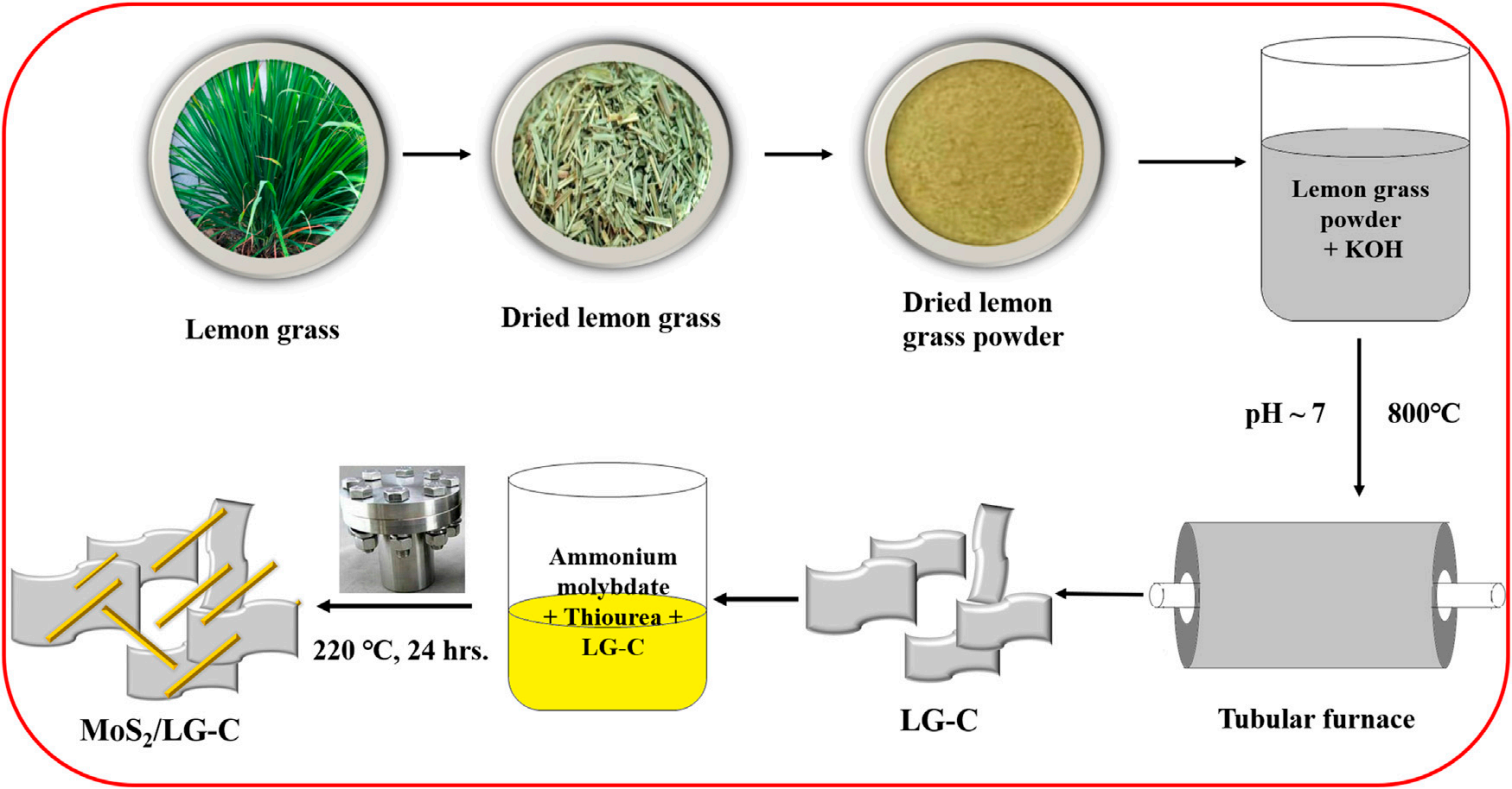
Schematic illustration of synthesis procedures for LG-C and MoS_2_/LG-C hybrid material.

**FIGURE 2:**
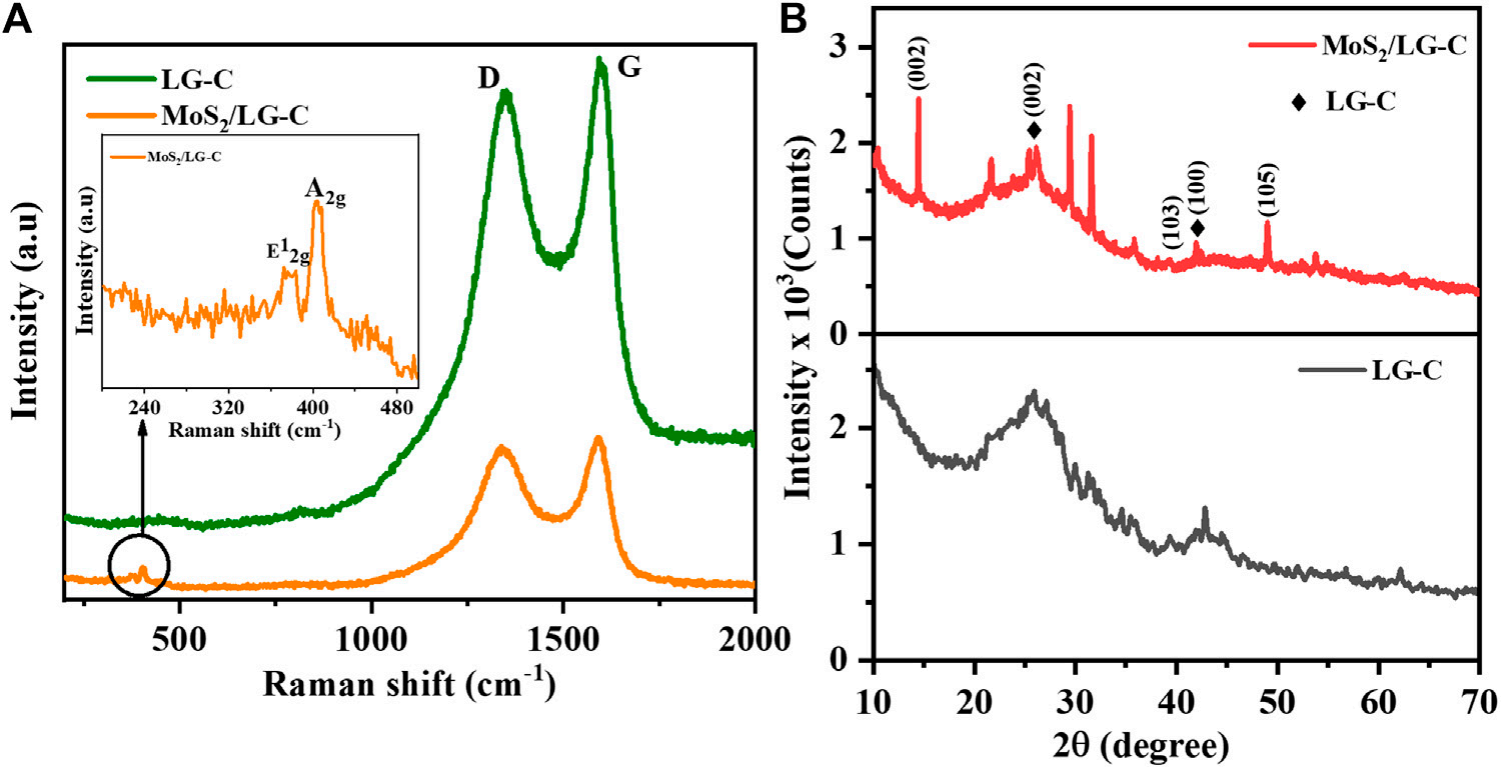
**(A)** Raman spectra **(B)** XRD pattern of LG-C and MoS_2_/LG-C hybrid material.

The one-step hydrothermal method was used to produce the MoS_2_/LG-C hybrid material. Figure 1 illustrates a schematic representation of the synthesis process of LG-C and MoS_2_/LG-C. LG-C nanosheets were associated with the formation of MoS_2_ nanobelts on the surfaces of LG-C. The structural information of MoS_2_ and the LG carbon was carried out using Raman spectroscopy and XRD analysis. Figure 2A shows the Raman spectra of LG-C and MoS_2_-LG-C. The two strong peaks appeared at about 1,344.3 and 1,592.8 cm^−1^ identified to the D and G vibrational modes of LG-C. The D band is associated with defects and disorders induced in sp^2^ carbon (Medhat et al., 2021). The Raman spectra of MoS_2_/LG-C show the E^1^_2g_ and A_1g_ vibrational modes, which conform to the layered 2H-MoS_2_ structure (Dinh et al., 2021). The peak observed at 376.2 and 404.4 cm^−1^ shown in the inset of Figure 2A corresponds to the E^1^_2g_ and A_1g_ modes of vibration containing in-plane and the plane-symmetric modes of Mo and S. The MoS_2_/LG-C Raman spectra revealed a shift in D and G vibrating modes occurring at 1,337.9 and 1,594.7 cm^−1^ which can be related to the stress caused in LG-C by the growth of MoS_2_ nanobelts. The intensity ratio of I_D_ to I_G_ is widely recognised to characterise the graphitization and amorphous nature of LG-C. In MoS_2_/LG-C, it was observed that the I_D/IG_ ratio was approximately similar to 1:1 (Kishore et al., 2014). This indicates that higher disorder and more active sites in MoS_2_ allowed for the formation of amorphous carbon and defective sites in MoS_2_/LG-C (Yu et al., 2020).

Figure 2B shows the XRD patterns of the as-synthesized LG-C and MoS_2_/LG-C hybrid materials. The diffraction peaks observed in MoS_2_/LG-C at 2θ values of 14.4°, 39.2°, and 49.0° have indexed the planes (002), (103), and (105) respectively, corresponding to the 2H hexagonal phase of MoS_2_ (JCPDS no. 37–1492). The pristine LG-C XRD pattern revealed two peaks at 26.0° and 42.7°. The broad XRD peak was obtained for the pristine LG-C, while in MoS_2_/LG-C, these peaks were found to be slightly shifted to 25.8° and 41.9° due to the presence of MoS_2_ on the surface of LG-C (Shapira and Zucker, 2022).

**FIGURE 3:**
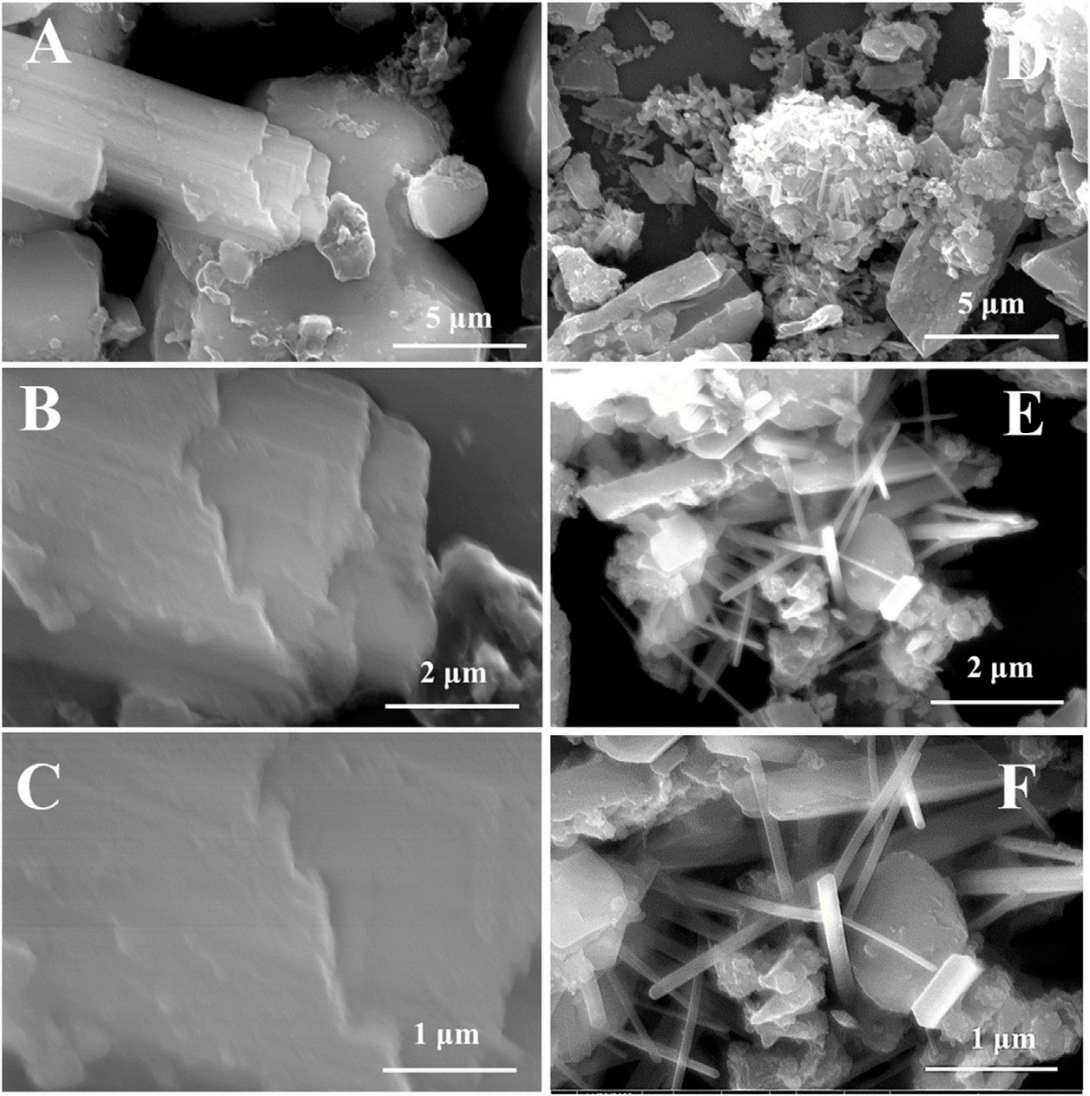
FESEM images **(A–C)** LG-C, and **(D–F)** MoS_2_/LG-C hybrid material.

FESEM images were taken at different magnifications, consisting of the micro-as well as nano-size surface morphology of pristine LG-C before and after the formation of MoS_2_. FESEM images of pristine LG-C, (Figures 3A–C) showed a thick sheet with a scattered layer appearance. LG-C sheets revealed a rough surface with the ordered stacking of the sheets. MoS_2_ nanobelts distributed in the interlayer of the LG-C materials (Figures 3D–F) produce nanobelts-like morphology. MoS_2_ has several precise nanobelts of sizes ranging from 2 to 3 μm in length with smooth and even surfaces.

**FIGURE 4:**
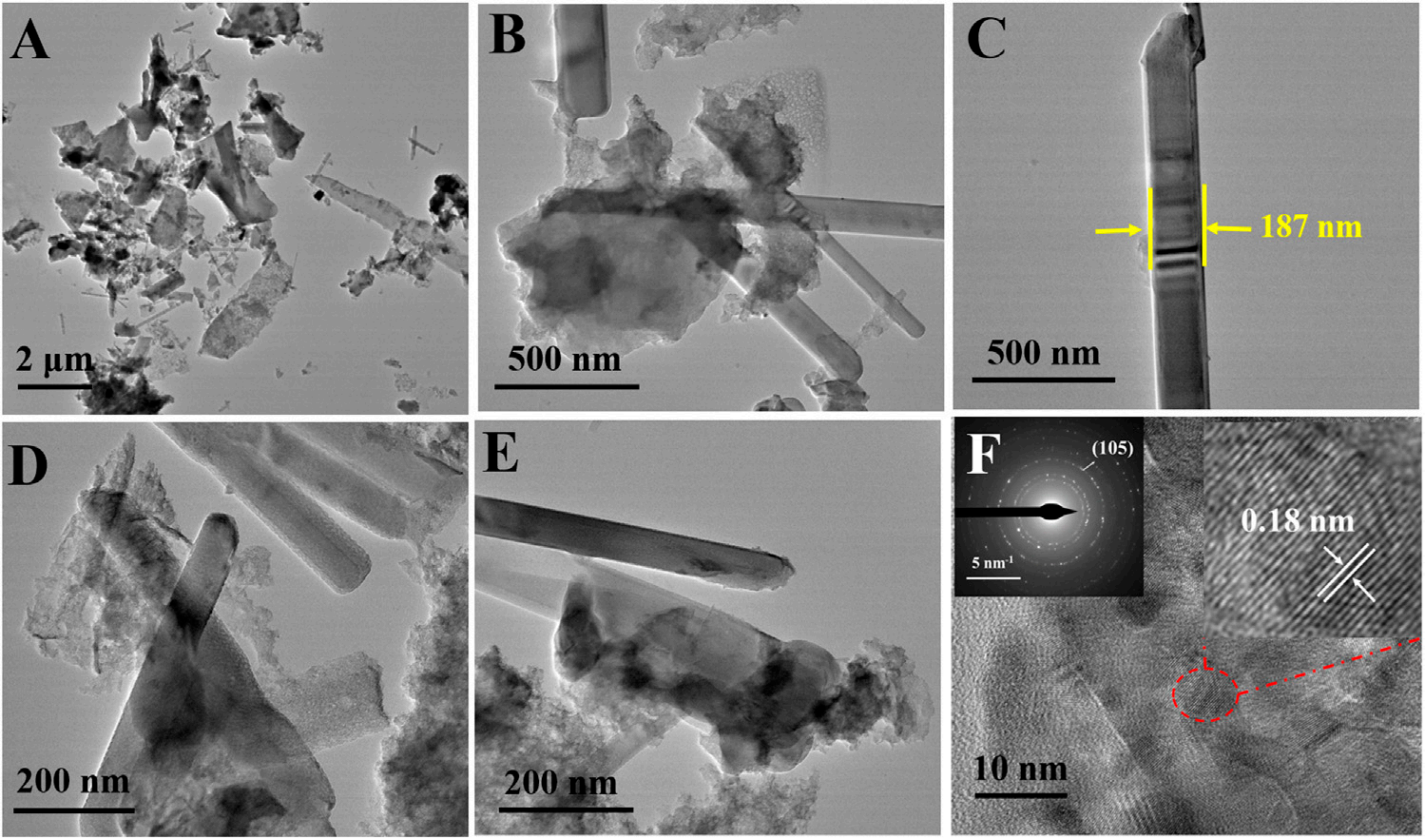
MoS_2_/LG-C hybrid material: **(A–E)** TEM images **(F)** HR-TEM image, inset of **(F)** showing SAED pattern and selected portion of lattice fringes.

Further, the surface morphology was investigated by TEM and HR-TEM images. The TEM images shown in Figures 4A–E further conform the size of nanobelts is not uniform, which varies from 90 to 190 nm. A clear dispersion of the MoS_2_ nanobelts in the LG-C matrix can be seen. The HR-TEM image (Figure 4F) shows a lattice spacing of ∼0.18 nm, which corresponds to the (105) plane of the hexagonal crystal structure of MoS_2,_ which matches with the XRD results. The selected-area electron diffraction (SAED) pattern of the MoS_2_/LG-C is shown in the inset of Figure 4F. SAED pattern exhibited the polycrystalline nature of MoS_2_/LG-C hybrid material is revealed. Some of the prominent reflections (105) are shown in SAED. The elemental composition determined with the help of EDS analysis is given in (Supplementary Figure S1). The observed composition of Mo and S was found to close to the ideal conditions (1:2).

**FIGURE 5:**
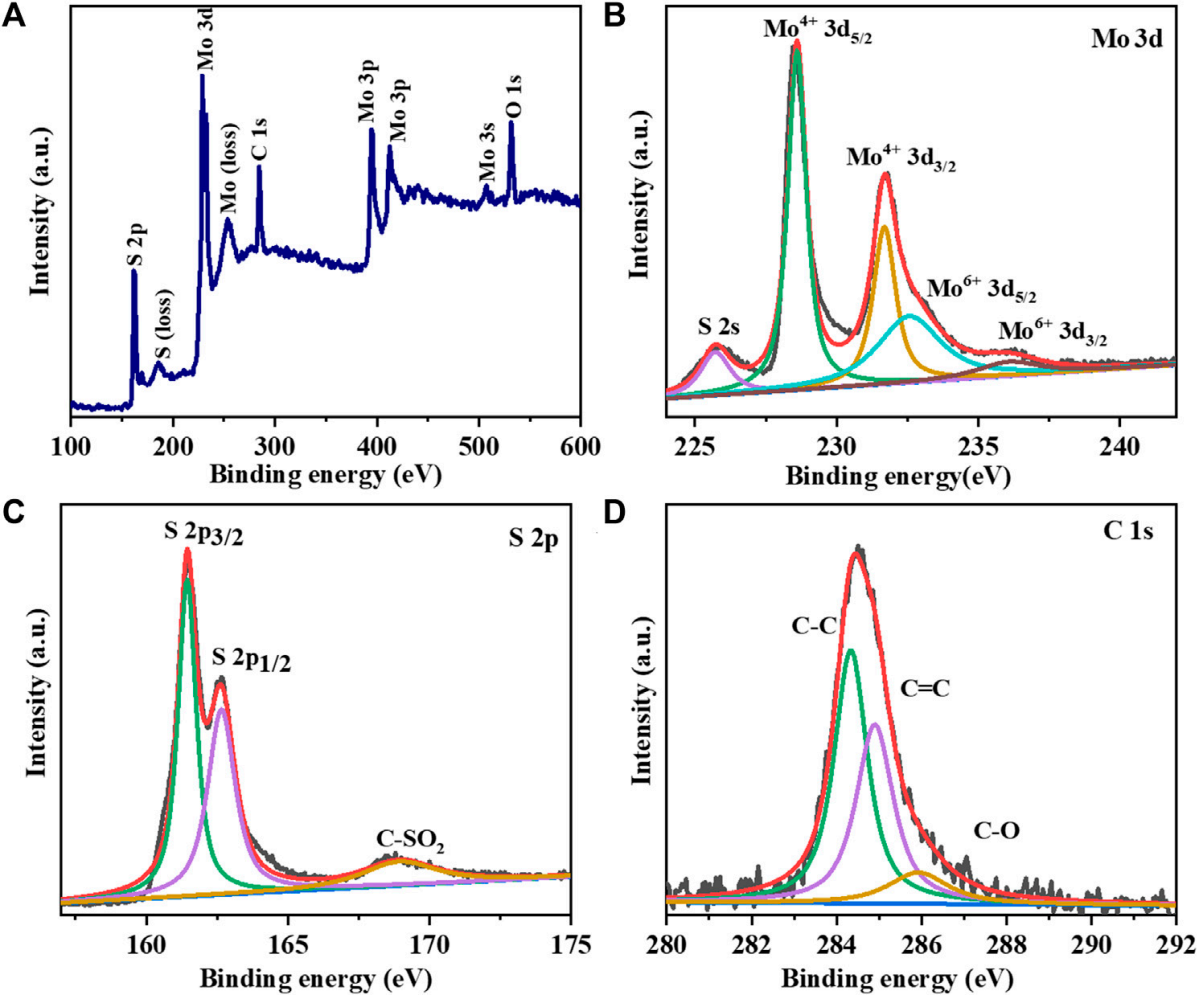
XPS analysis of MoS_2_/LG-C hybrid material: **(A)** Survey scan; High resolution XPS spectrum of **(B)** Mo 3d **(C)** S 2p **(D)** C 1s.

The XPS spectrum of the MoS_2_/LG-C hybrid material is depicted in Figure 5. Figure 5A shows the survey scan for four peaks conforming to Mo 3d, S 2p, C 1s, and O 1s, suggesting that the MoS_2_/LG-C hybrid material was successfully formed. Apart from them, the peaks were obtained at 186.0 and 253.4 eV called plasmon loss peaks of S and Mo respectively. These peaks occur may be a higher probability of losing a specified amount of energy as a result of the photoelectron’s interaction with other electrons (Stevie and Donley, 2020). The doublet obtained 394.8 and 412.6 eV corresponds to Mo 3p_3/2_ and Mo 3p_½_ respectively. The Mo 3d XPS resolution scan (Figure 5B) shows the two primary peaks were obtained at 228.6 and 231.7 eV, corresponding to Mo^4+^ 3d_5/2_ and Mo^4+^ 3d_3/2_, respectively (Feng et al., 2019). The peaks obtained at 232.6 eV and 236.2 eV correspond to Mo^6+^ 3d_5/2_ and Mo^6+^ 3d_3/2,_ respectively. The existence of sulphur atom level 2s in the MoS_2_/LG-C was linked to the peak at 225.7 eV (Xiong et al., 2015). Figure 5C shows the high-resolution S 2p XPS spectra, indicating the presence of peaks conforming to the S 2p_3/2_ and S 2p_1/2_ situated at 161.5 and 162.5 eV. The peak attributed to 168.9 eV could be the presence of the sulphate group (Gnanasekar et al., 2020). The C1s XPS spectra in Figure 5D revealed three major peaks with binding energies of about 284.3, 284.8, and 286.1, eV, corresponding to the functional groups C-C, C-C, and C-O, respectively (Ji et al., 2018).

**FIGURE 6:**
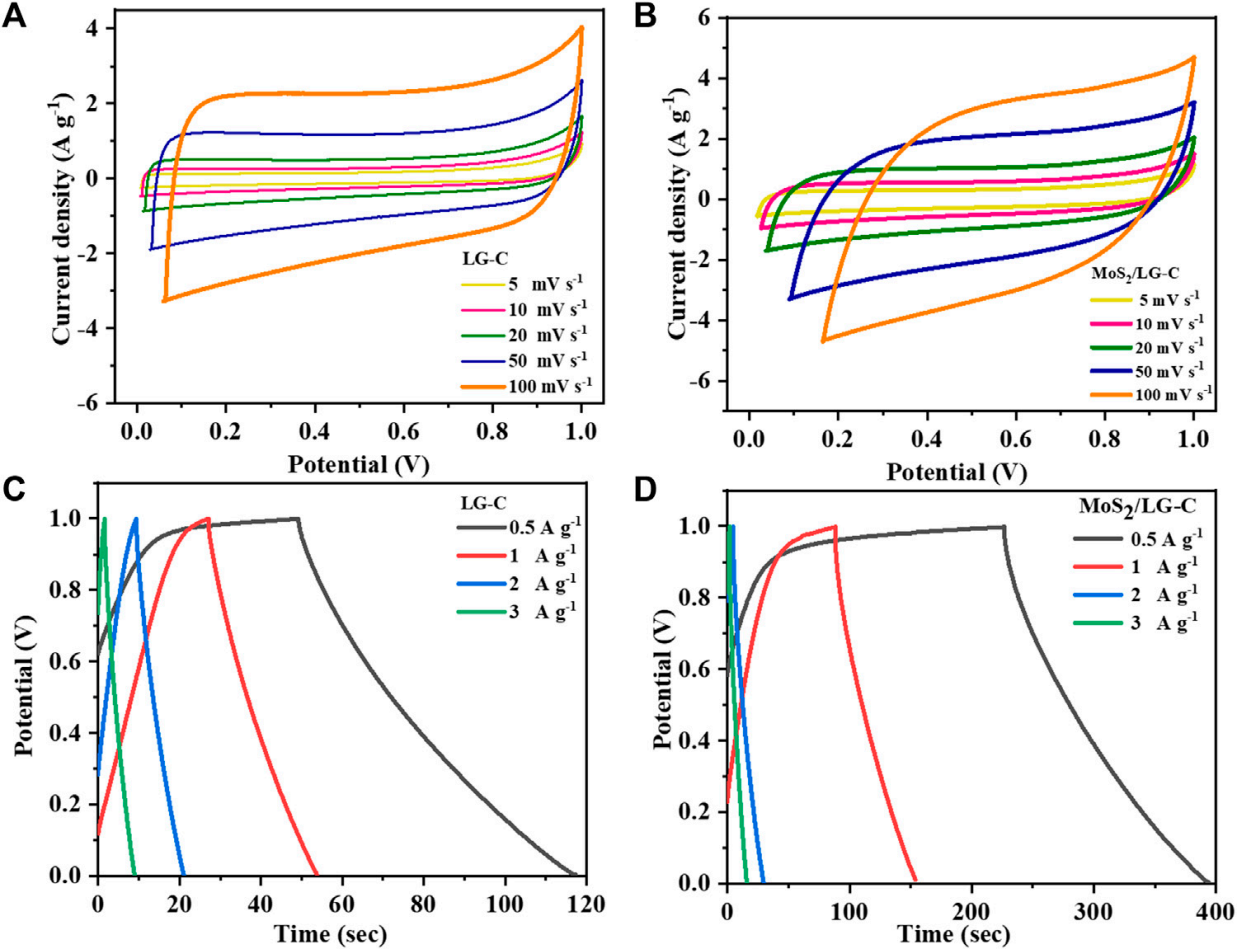
CV curves of **(A)** LG-C, **(B)** MoS_2_/LG-C; GCD curves of **(C)** LG-C, **(D)** MoS_2_/LG-C obtained by 3-electrode system.

In Figure 6, all CV and GCD measurements were carried out using a 3-electrode system. Figures 6A, B shows the CV curves of LG-C and MoS_2_/LG-C in 1 M Li_2_SO_4_ at scan rates of 5, 10, 20, 50, and, 100 mV s^−1^. The CV curve of the LG-C electrode (Figure 6A), shows an almost rectangular nature with no identifiable redox peaks in the operating voltage range of 0.0–1.0 V. The rectangular nature of the CV curve of LG-C exhibits ideal behavior, showing the electric double-layer capacitor (Luo et al., 2015). MoS_2_/LG-C hybrid material in Figure 6B has a dome-like quasi-rectangular shape that exhibits both electric double-layer capacitor behaviour as well as Faradic pseudocapacitance behaviour (Xu et al., 2018). MoS_2_ nanobelts distributed in the interlayer of the LG-C sheets increased the entire area of the CV as well as peak current density with improved conductivity, which helps facilitate ion transport towards the electrochemical charge storage (Gopalakrishnan et al., 2020; Mahajan et al., 2022).

Figures 6C, D show the GCD curves of LG-C and MoS_2_/LG-C at current densities of 0.5, 1, 2, and 3 A g^−1^ with the applied potential voltage between 0.0 and 1.0 V. The specific capacitance (Cs in F g^−1^) was calculated at various current densities using the following Eq. 1 (Lin and Zhang, 2015):

$$C_{s}=\frac{I∆t}{m∆V}$$ (1)

where I is the current density, ∆V is the applied potential window, m is the active mass of the electrode, and ∆t is discharge time.

The Cs of LG-C and MoS_2_/LG-C calculate to be 30.5 F g^−1^ and 77.5 F g^−1^ at a current density of 0.5 A g^−1^, respectively. MoS_2_/LG-C helps to increase the specific capacitance value of the hybrid electrode material due to the synergistic effects of MoS_2_ as a conducting material with LG-C. Mo^4+^ in MoS_2_ supplied the more active sites for charge transfer in MoS_2_/LG-C, which provided a more interactive area between MoS_2_/LG-C and the electrolyte for ion diffusion and increased the discharge time (Feng et al., 2019). The comparative CV and GCD curves of LG-C and MoS2/LG-C are shown in Supplementary Figures S2A, B at a scan rate of 20 mV s^−1^ and a current density of 0.5 A g^−1^. Electrochemical charge storage is obtained due to ions present in an electrolyte (Li^+^) interaction through MoS_2_ and carbon sheets.

**FIGURE 7:**
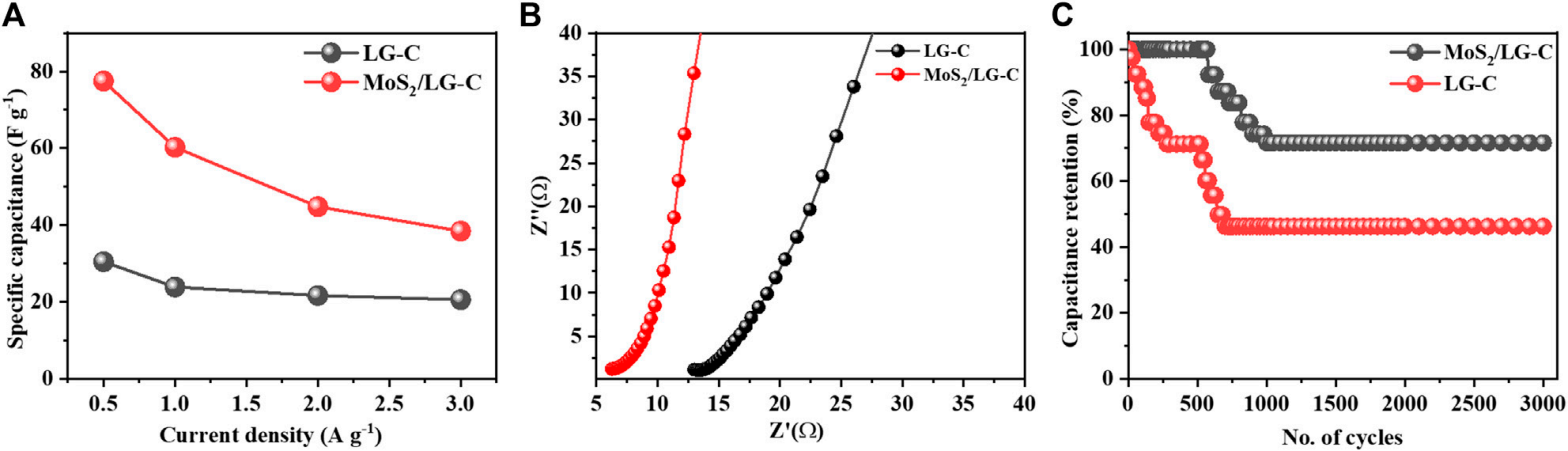
**(A)** Comparative plot of Cs at different current densities, **(B)** Nyquist plot of LG-C and MoS_2_/LG-C at the frequency range from 0.1 MHz to 100 mHz, **(C)** cyclic stability plot of LG-C and MoS_2_/LG-C hybrid material at current density 3 A g^−1^.

Figure 7A illustrates the plot of C_s_ *versus* the different current densities. At higher current densities, C_s_ were found to decrease, which could be due to the reduction in various activities at the electrode surface. The capacitance improvement occurs due to the favourable synergistic effect of MoS_2_ as an active-site hybrid material, which helps to avoid the stacking of LG-C sheets and increases the surface area of MoS_2_/LG-C. The increased surface area provided the path for electrochemical charge transformation and helped store the energy in the supercapacitor (Bi et al., 2019). The comparative data of different composite nanostructure materials with MoS_2_/LG-C in the literature related to Cs and various electrolytes are shown in Table 1. (Brousse et al., 2007; Aradilla et al., 2014; Ho et al., 2014; Masikhwa et al., 2017; Choi et al., 2020; Bello et al., 2022). The EIS measurements of LG-C and MoS_2_/LG-C were performed to study the transportation of ions in electrolytes with their electrochemical properties, equivalent series resistance (R_s_), and charge transfer resistance (R_ct_) (Dulyaseree et al., 2017). Figure 7B shows the Nyquist plot of LG-C and MoS_2_/LG-C hybrid materials with an imaginary axis performed at a frequency range of 0.1 MHz–100 mHz. LG-C and MoS_2_/LG-C achieved similar series resistance (R_s_) for supercapacitors of about 12.09 Ω and 6.26 Ω which shows the MoS_2_/LG-C has good electrical conductivity. In the high-frequency region, a clear semicircle was not observed, which indicates it may have a low R_ct_ (Niaz et al., 2020). MoS_2_/LG-C has increased the conductivity of hybrid material because MoS_2_ provided a more active site for LG-C, which helps improve electrochemical properties. The cyclic stability of LG-C and MoS_2_/LG-C (Figure 7C was carried out using a GCD test in 1M Li_2_SO_4_ electrolyte at the current density of 3 A g^−1^. MoS_2_/LG-C exhibited superior cyclic stability and, 71.6% capacitance retaliation, and only 28.4% capacity degradation occurs up to 3,000 cycles at a current density of 3 A g^−1^. In LG-C, 46.2% of the capacitance was retained up to 3,000 cycles. In MoS_2_/LGC hybrid materials, sulphur bonded with MoS_2_ forms a layer that is collected on the carbon surface and connected to the carbon structure covalently, which helps to provide excellent cyclic stability. The abrupt decrease in capacity observed in cyclic stability plot due to the measurement might be related to active material dissolving in the electrolyte, as well as difficulties with electrodes which include expansion and active material loss owing to poor binding.

**TABLE 1:** Comparative electrochemical performance of various nanostructured materials.

Sr No	Nanostructure materials	Cs	Current density/Scan rate	Electrolyte	Ref
1	Activated Carbon/MnO_2_ Composites	60.3 F g^-1^	1 A g^-1^	1 M Na_2_SO_4_	Choi et al. (2020)
2	Mixed-Phase Mn-Doped MoS_2_ Nanoflower	70.37 F g^-1^	1A g^-1^	1 M Na_2_SO_4_	Bello et al. (2022)
3	activated carbon/MnO_2_	23 F g^-1^	2 mV s^-1^	Aq. K_2_SO_4_	Brousse et al. (2007)
4	Nano Fe_3_ O_4_ - Activated Carbon Composites	42.88 F g^-1^	10 mV s^-1^	1M Na_2_SO_3_	Ho et al. (2014)
5	MoS_2_/graphene foam composites	59 F g^-1^	1A g^-1^	6M KOH	Masikhwa et al. (2017)
6	PEDOT on Si nanowires	32 F g^-1^	0.1 mA cm^-2^	PYR_13_TFSI	Aradilla et al. (2014)
7	MoS_2_/LG-C	77.5 F g^-1^	0.5 A g^-1^	1 M Li_2_SO_4_	Present work

**FIGURE 8:**
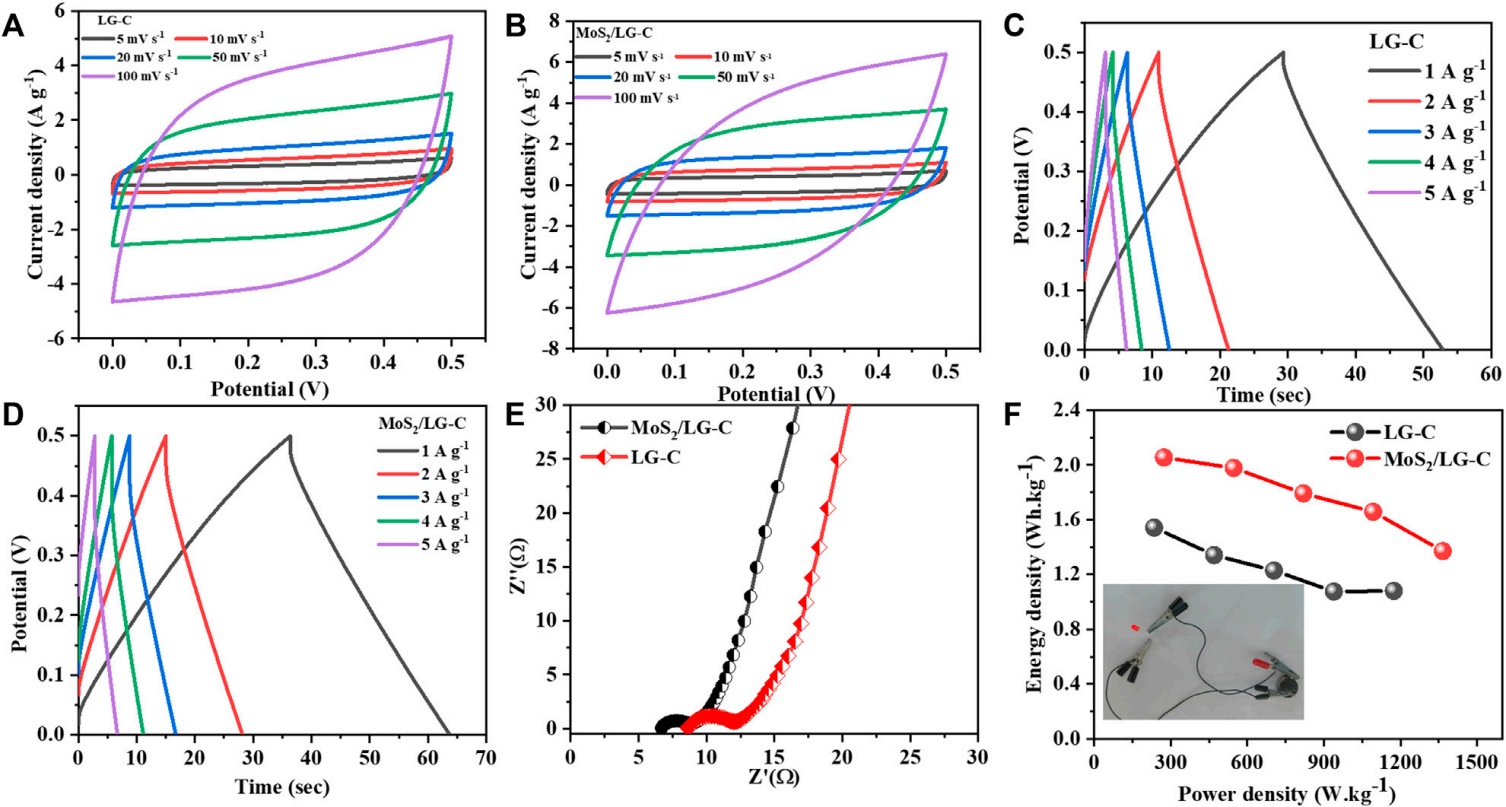
Symmetric supercapacitor device electrochemical measurements: **(A)** CV curves of LG-C, **(B)** CV curves of MoS_2_/LG-C, **(C)** GCD curves of LG-C, **(D)** GCD curves of MoS_2_/LG-C, **(E)** Nyquist plot of LG-C and MoS_2_/LG-C, **(F)** Ragone plot of LG-C and MoS_2_/LG-C, Inset of **(F)** shows the operating red light emitting diode for symmetric supercapacitor devices.

The electrochemical behaviour of the LG-C and MoS_2_/LG-C hybrid nanomaterials were also studied by symmetric supercapacitor devices in 1M Li_2_SO_4_ electrolyte. The CV curves of LG-C and MoS_2_/LG-C (Figures 8A, B) were measured at the potential window of 0.0–0.5 V at scan rates between 5 and 100 mVs^−1^. All CV curves of LG-C have a quasi-rectangular shape, showing a good electric double-layer capacitor for fast ion transfer. While CV curves variations from an ideal rectangle with increasing the current density. The GCD measurements of LG-C and MoS_2_/LG-C exhibit minor MoS_2_/LG-C were carried out at different current densities are shown in Figures 8C, D, respectively. The charge-discharge durations of the MoS_2_/LG-C hybrid material has longer than those of pure LG-C. The MoS_2_/LG-C hybrid material has higher specific capacitance than pure LG-C. The specific capacitance values were calculated using Eq. 2 for symmetric supercapacitor devices.

$$C_{s}=2\left(\frac{I∆t}{m∆V}\right)$$ (2)

The MoS_2_/LG-C hybrid material and LG-C show a specific capacitance of 59.2 F g^−1^and 44.4 F g^−1^, respectively at a current density of 1 A g^−1^.

Furthermore, EIS measurements of MoS_2_/LG-C hybrid material and LG-C (Figure 8E) were carried out in the frequency range of 100 kHz to 10 mHz. The Nyquist plot corresponding series resistance (R_s_) and charge transfer resistance (R_ct_) values of LG-C are 8.5 and 3.4 Ω respectively. In the MoS_2_/LG-C Nyquist plot, Rs and R_ct_ were determined to be 6.6 and 2.2 Ω, respectively. The lower R_s_ value implies the internal resistance, electrolyte ionic resistance, and contact resistance of active electrode materials of the MoS_2_/LG-C electrode is more conductive than the LG-C electrode. The R_ct_ may be ascribed to the MoS_2_/LG-C electrode’s improved electron transportation and high surface area, which allows for quick redox reactions at the electrode/electrolyte interface.

The Ragone plot of LG-C and MoS_2_/LG-C obtained at various current densities is depicted in Figure 8F. The relation between energy density (E in Wh kg^−1^) and power density (P in W kg^−1^) is an important parameter to examine energy devices. Energy density and power density are calculated from the following equations: 3 and 4, respectively (Raghu et al., 2018).

$$E=\frac{1}{2}\;C_{s}{∆V}^{2}$$ (3)

$$P=\frac{E}{∆t}$$ (4)

The MoS_2_/LG-C-based supercapacitor performance measured 273.2 W kg^−1^ power density and 2.1 Wh kg^−1^ energy density, whereas the LG-C sheets-based supercapacitor exhibited 234.7 W kg^−1^ power density and 1.5 Wh kg^−1^ energy density at a current density of 1 A g^−1^. P and E were found to increase with increasing current density, and the maximum power density, 1,366.1 W kg^−1^, and energy density, 1.3 Wh kg^−1^ were obtained at a current density of 5 A g^−1^ for MoS_2_/LG-C symmetric device. The inset of Figure 8F shows the operating red light-emitting diode for symmetric supercapacitor devices.

## Conclusion

In conclusion, we successfully synthesized the MoS_2_ Nanobelts/LG-C nanohybrid structures by simple hydrothermal method. The low-cost and biodegradable LG leaves were used to form the nano-carbon by the pyrolysis method. The MoS_2_ nanobelts help to provide pathways for ion transportation due to the interaction between active electrode materials and electrolytes. The specific capacitances of MoS_2_/LG-C and LG-C were found to be 77.5 and 30.1 F g^−1^, respectively, at a current density of 0.5 A g^−1^. The cyclic stability of MoS_2_/LG-C and LG-C was carried out by the GCD test, and 71.6% capacitance retention occurs up to 3,000 cycles at the current density of 3 A g^−1^. The symmetric supercapacitor (MoS_2_/LG-C//MoS_2_/LG-C), the operating red light-emitting diode is illuminated. This method is favourable for the large-scale production of MoS_2_/LGC as active electrode hybrid materials for electrochemical energy storage devices.

## Data Availability

The original contributions presented in the study are included in the article/Supplementary Material.

## References

[B1] Agrawal A. Siddiqui S. A. Soni A. Sharma G. D. (2022). Advancements, frontiers and analysis of metal oxide semiconductor, dye, electrolyte and counter electrode of dye sensitized solar cell. Sol. Energy 233. 10.1016/j.solener.2022.01.027

[B2] Aradilla D. Bidan G. Gentile P. Weathers P. Thissandier F. Ruiz V. (2014). Novel hybrid micro-supercapacitor based on conducting polymer coated silicon nanowires for electrochemical energy storage. Rsc Adv. 4. 10.1039/c4ra03192j

[B3] Atta M. M. Fahim R. A. (2021). Flexible and wearable supercapacitors: A short review. J. Energy Storage 44, 103475. 10.1016/j.est.2021.103475

[B4] Bai S. Wang T. Tian Z. Cao K. Li J. (2020). Facile preparation of porous biomass charcoal from peanut shell as adsorbent. Sci. Rep. 10. 10.1038/s41598-020-72721-0 PMC7522232 32985585

[B5] Bello I. T. Otun K. O. Nyongombe G. Adedokun O. Kabongo G. L. Dhlamini M. S. (2022). Synthesis, characterization, and supercapacitor performance of a mixed-phase Mn-doped MoS2 nanoflower. Nanomaterials 12, 490. 10.3390/nano12030490 35159835 PMC8839322

[B6] Bi Z. Kong Q. Cao Y. Sun G. Su F. Wei X. (2019). Biomass-derived porous carbon materials with different dimensions for supercapacitor electrodes: A review. J. Mat. Chem. a 7. 10.1039/c9ta04436a

[B7] Brousse T. Taberna P.-L. Crosnier O. Dugas R. Guillemet P. Scudeller Y. (2007). Long-term cycling behavior of asymmetric activated carbon/MnO2 aqueous electrochemical supercapacitor. J. Power Sources 173. 10.1016/j.jpowsour.2007.04.074

[B8] Choi J. R. Lee J. W. Yang G. Heo Y.-J. Park S.-J. (2020). Activated carbon/MnO2 composites as electrode for high performance supercapacitors. Catalysts 10, 256. 10.3390/catal10020256

[B9] Cook J. B. Kim H. Lin T. C. Lai C. Dunn B. Tolbert S. H. (2017). Pseudocapacitive charge storage in thick composite MoS2 nanocrystal‐based electrodes. Adv. Energy Mat. 7, 1601283. 10.1002/aenm.201601283

[B10] Dinh D. A. Nguyen T. L. Cuong T. V. Hui K. S. Bui T. H. Wu S. (2021). Defect-free MoS2-flakes/amorphous-carbon hybrid as an advanced anode for lithium-ion batteries. Energy & Fuels 35. 10.1021/acs.energyfuels.0c03896

[B11] Dulyaseree P. Fujishige M. Yoshida I. Toya Y. Banba Y. Tanaka Y. (2017). Nitrogen-rich green leaves of papaya and Coccinia grandis as precursors of activated carbon and their electrochemical properties. RSC Adv. 7. 10.1039/c7ra06048c,

[B12] Feng N. Meng R. Zu L. Feng Y. Peng C. Huang J. (2019). A polymer-direct-intercalation strategy for MoS2/carbon-derived heteroaerogels with ultrahigh pseudocapacitance. Nat. Commun. 10. 10.1038/s41467-019-09384-7 PMC6435689 30914649

[B13] Fleischmann S. Zhang Y. Wang X. Cummings P. T. Wu J. Simon P. (2022). Continuous transition from double-layer to Faradaic charge storage in confined electrolytes. Nat. Energy 7. 10.1038/s41560-022-00993-z

[B14] Gaddam R. R. Yang D. Narayan R. Raju K. Kumar N. A. Zhao X. S. (2016). Biomass derived carbon nanoparticle as anodes for high performance sodium and lithium ion batteries. Nano energy 26. 10.1016/j.nanoen.2016.05.047

[B15] Gnanasekar P. Ranjith K. S. Manivel P. Han Y.-K. Kulandaivel J. (2020). Hierarchical NbS2/MoS2-carbon nanofiber electrode for highly efficient and stable hydrogen evolution reaction at all ranges of pH. ACS Appl. Energy Mat. 3. 10.1021/acsaem.0c00856

[B16] Gomes Ferreira de Paula F. Campello-Gómez I. Ortega P. F. R. Rodríguez-Reinoso F. Martínez-Escandell M. Silvestre-Albero J. (2019). Structural flexibility in activated carbon materials prepared under harsh activation conditions. Mater. (Basel) 12, 1988. 10.3390/ma12121988 PMC6632014 31226832

[B17] Gopalakrishnan A. Yu A. Badhulika S. (2020). Facile synthesis of highly porous N-doped carbon nanosheets with silica nanoparticles for ultrahigh capacitance supercapacitors. Energy & Fuels 34. 10.1021/acs.energyfuels.0c02078

[B18] Ho M. Y. Khiew P. S. Isa D. Tan T. K. Chiu W. S. Chia C. H. (2014). Nano Fe3O4-activated carbon composites for aqueous supercapacitors. Sains Malays. 43, 885–894.

[B19] Huang S. Zhu X. Sarkar S. Zhao Y. (2019). Challenges and opportunities for supercapacitors. Apl. Mater 7, 100901. 10.1063/1.5116146

[B20] Iro Z. S. Subramani C. Dash S. S. (2016). A brief review on electrode materials for supercapacitor. Int. J. Electrochem. Sci. 11. 10.20964/2016.12.50

[B21] Ji H. Hu S. Shi S. Guo B. Hou W. Yang G. (2018). Rapid microwave-hydrothermal preparation of few-layer MoS2/C nanocomposite as anode for highly reversible lithium storage properties. J. Mat. Sci. 53. 10.1007/s10853-018-2631-7

[B22] Kandasamy M. Sahoo S. Nayak S. K. Chakraborty B. Rout C. S. (2021). Recent advances in engineered metal oxide nanostructures for supercapacitor applications: Experimental and theoretical aspects. J. Mat. Chem. A 9. 10.1039/d1ta03857e

[B23] Khandare L. Late D. J. (2017). MoO3-rGO nanocomposites for electrochemical energy storage. Appl. Surf. Sci. 418. 10.1016/j.apsusc.2016.11.199

[B24] Kishore B. Shanmughasundaram D. Penki T. R. Munichandraiah N. (2014). Coconut kernel-derived activated carbon as electrode material for electrical double-layer capacitors. J. Appl. Electrochem. 44. 10.1007/s10800-014-0708-9

[B25] Kour S. Tanwar S. Sharma A. L. (2022). MnO2 nanorod loaded activated carbon for high-performance supercapacitors. J. Alloys Compd. 910, 164834. 10.1016/j.jallcom.2022.164834

[B26] Kukkar M. Tuteja S. K. Sharma A. L. Kumar V. Paul A. K. Kim K.-H. (2016). A new electrolytic synthesis method for few-layered MoS2 nanosheets and their robust biointerfacing with reduced antibodies. ACS Appl. Mat. Interfaces 8. 10.1021/acsami.6b03079 27296984

[B27] Li X. Zhang J. Liu B. Su Z. (2021). A critical review on the application and recent developments of post-modified biochar in supercapacitors. J. Clean. Prod. 310, 127428. 10.1016/j.jclepro.2021.127428

[B28] Liakos I. L. D’autilia F. Garzoni A. Bonferoni C. Scarpellini A. Brunetti V. (2016). All natural cellulose acetate—lemongrass essential oil antimicrobial nanocapsules. Int. J. Pharm. 510. 10.1016/j.ijpharm.2016.01.060 26827919

[B29] Lin S.-Y. Zhang X. (2015). Two-dimensional titanium carbide electrode with large mass loading for supercapacitor. J. Power Sources 294. 10.1016/j.jpowsour.2015.06.082

[B30] Luo Q.-P. Huang L. Gao X. Cheng Y. Yao B. Hu Z. (2015). Activated carbon derived from melaleuca barks for outstanding high-rate supercapacitors. Nanotechnology 26, 304004. 10.1088/0957-4484/26/30/304004 26152815

[B31] Luo X. Chen S. Hu T. Chen Y. Li F. (2021). Renewable biomass‐derived carbons for electrochemical capacitor applications. SusMat 1. 10.1002/sus2.8

[B32] Madhu R. Sankar K. V. Chen S.-M. Selvan R. K. (2014). Eco-friendly synthesis of activated carbon from dead mango leaves for the ultrahigh sensitive detection of toxic heavy metal ions and energy storage applications. Rsc Adv. 4. 10.1039/c3ra45089a,

[B33] Mahajan H. Mohanan K. U. Cho S. (2022). Facile synthesis of biocarbon-based MoS2 composite for high-performance supercapacitor application. Nano Lett. 22. 10.1021/acs.nanolett.2c02595, PMC9614961 36194392

[B34] Mahmood Q. Park S. K. Kwon K. D. Chang S. Hong J. Shen G. (2016). Transition from diffusion‐controlled intercalation into extrinsically pseudocapacitive charge storage of MoS2 by nanoscale heterostructuring. Adv. Energy Mat. 6, 1501115. 10.1002/aenm.201501115

[B35] Masikhwa T. M. Madito M. J. Bello A. Dangbegnon J. K. Manyala N. (2017). High performance asymmetric supercapacitor based on molybdenum disulphide/graphene foam and activated carbon from expanded graphite. J. Colloid Interface Sci. 488. 10.1016/j.jcis.2016.10.095 27825060

[B36] Mathis T. S. Kurra N. Wang X. Pinto D. Simon P. Gogotsi Y. (2019). Energy storage data reporting in perspective—Guidelines for interpreting the performance of electrochemical energy storage systems. Adv. Energy Mat. 9, 1902007. 10.1002/aenm.201902007

[B37] Medhat A. El-Maghrabi H. H. Abdelghany A. Menem N. M. A. Raynaud P. Moustafa Y. M. (2021). Efficiently activated carbons from corn cob for methylene blue adsorption. Appl. Surf. Sci. Adv. 3, 100037. 10.1016/j.apsadv.2020.100037

[B38] Mohammed A. A. Chen C. Zhu Z. (2019). Low-cost, high-performance supercapacitor based on activated carbon electrode materials derived from baobab fruit shells. J. Colloid Interface Sci. 538. 10.1016/j.jcis.2018.11.103 30530028

[B39] Narthana K. Durai G. Kuppusami P. Theerthagiri J. Sujatha S. Lee S. J. (2021). One‐step synthesis of hierarchical structured nickel copper sulfide nanorods with improved electrochemical supercapacitor properties. Int. J. Energy Res. 45. 10.1002/er.6492

[B40] Niaz N. A. Shakoor A. Imran M. Khalid N. R. Hussain F. Kanwal H. (2020). Enhanced electrochemical performance of MoS2/PPy nanocomposite as electrodes material for supercapacitor applications. J. Mat. Sci. Mat. Electron. 31. 10.1007/s10854-020-03682-3

[B41] Purkait T. Singh G. Kumar D. Singh M. Dey R. S. (2018). High-performance flexible supercapacitors based on electrochemically tailored three-dimensional reduced graphene oxide networks. Sci. Rep. 8. 10.1038/s41598-017-18593-3 PMC5766552 29330476

[B42] Raghu M. S. Kumar K. Y. Rao S. Aravinda T. Prasanna B. P. Prashanth M. K. (2018). Fabrication of polyaniline–few-layer MoS2 nanocomposite for high energy density supercapacitors. Polym. Bull. 75. 10.1007/s00289-017-2267-9

[B43] Rajagopal S. Pulapparambil Vallikkattil R. Mohamed Ibrahim M. Velev D. G. (2022). Electrode materials for supercapacitors in hybrid electric vehicles: Challenges and current progress. Condens. Matter 7, 6. 10.3390/condmat7010006

[B44] Rawat S. Mishra R. K. Bhaskar T. (2022). Biomass derived functional carbon materials for supercapacitor applications. Chemosphere 286, 131961. 10.1016/j.chemosphere.2021.131961 34426294

[B45] Ruan C. Ai K. Lu L. (2014). Biomass-derived carbon materials for high-performance supercapacitor electrodes. Rsc Adv. 4. 10.1039/c4ra04470c,

[B46] Santoro C. Winfield J. Theodosiou P. Ieropoulos I. (2019). Supercapacitive paper based microbial fuel cell: High current/power production within a low cost design. Bioresour. Technol. Rep. 7, 100297. 10.1016/j.biteb.2019.100297 31853518 PMC6894309

[B47] Shapira K. Zucker I. (2022). Emerging investigator series: Molybdenum disulfide-enabled activated carbon-a multifunctional adsorbent for practical water treatment applications. Environ. Sci. Nano 9. 10.1039/d1en00897h

[B48] Shaqsi A. Z. A. L. Sopian K. Al-Hinai A. (2020). Review of energy storage services, applications, limitations, and benefits. Energy Rep. 6. 10.1016/j.egyr.2020.07.028

[B49] Stevie F. A. Donley C. L. (2020). Introduction to x-ray photoelectron spectroscopy. J. Vac. Sci. Technol. A Vac. Surfaces, Film. 38, 063204. 10.1116/6.0000412

[B50] Thota S. P. Thota S. M. Srimadh Bhagavatham S. Sai Manoj K. Sai Muthukumar V. S. Venketesh S. (2018). Facile one-pot hydrothermal synthesis of stable and biocompatible fluorescent carbon dots from lemon grass herb. IET Nanobiotechnology 12. 10.1049/iet-nbt.2017.0038

[B51] Tomboc G. M. Tesfaye Gadisa B. Jun M. Chaudhari N. K. Kim H. Lee K. (2020). Carbon transition-metal oxide electrodes: Understanding the role of surface engineering for high energy density supercapacitors. Chem. Asian J. 15. 10.1002/asia.202000324 32301268

[B52] Tomy M. Ambika Rajappan A. Vm V. Thankappan Suryabai X. (2021). Emergence of novel 2D materials for high-performance supercapacitor electrode applications: A brief review. Energy & Fuels 35. 10.1021/acs.energyfuels.1c02743

[B53] Volfkovich Y. M. (2021). Electrochemical supercapacitors (a review). Russ. J. Electrochem. 57. 10.1134/s1023193521040108

[B54] Wang F. Ma J. Zhou K. Li X. (2020). MoS2/corncob-derived activated carbon for supercapacitor application. Mat. Chem. Phys. 244, 122215. 10.1016/j.matchemphys.2019.122215

[B55] Xiong X. Luo W. Hu X. Chen C. Qie L. Hou D. (2015). Flexible membranes of MoS2/C nanofibers by electrospinning as binder-free anodes for high-performance sodium-ion batteries. Sci. Rep. 5. 10.1038/srep09254 PMC5380159 25806866

[B56] Xu W. Mu B. Wang A. (2018). All-solid-state high-energy asymmetric supercapacitor based on natural tubular fibers. J. Mat. Sci. 53. 10.1007/s10853-018-2418-x

[B57] Yu X. Han X. Chang C. Hu Y. Xu C. C. Fang S. (2020). Corncob-derived activated carbon for roxarsone removal from aqueous solution: Isotherms, kinetics, and mechanism. Environ. Sci. Pollut. Res. 27. 10.1007/s11356-020-07942-x 32088818

