# Supplementary figures and images for: MoS2 nanobelts-carbon hybrid material for supercapacitor applications
Source: Front Chem. 2023 Aug 22;11:1166544. doi: 10.3389/fchem.2023.1166544 (PMC10477701; doi:10.3389/fchem.2023.1166544)

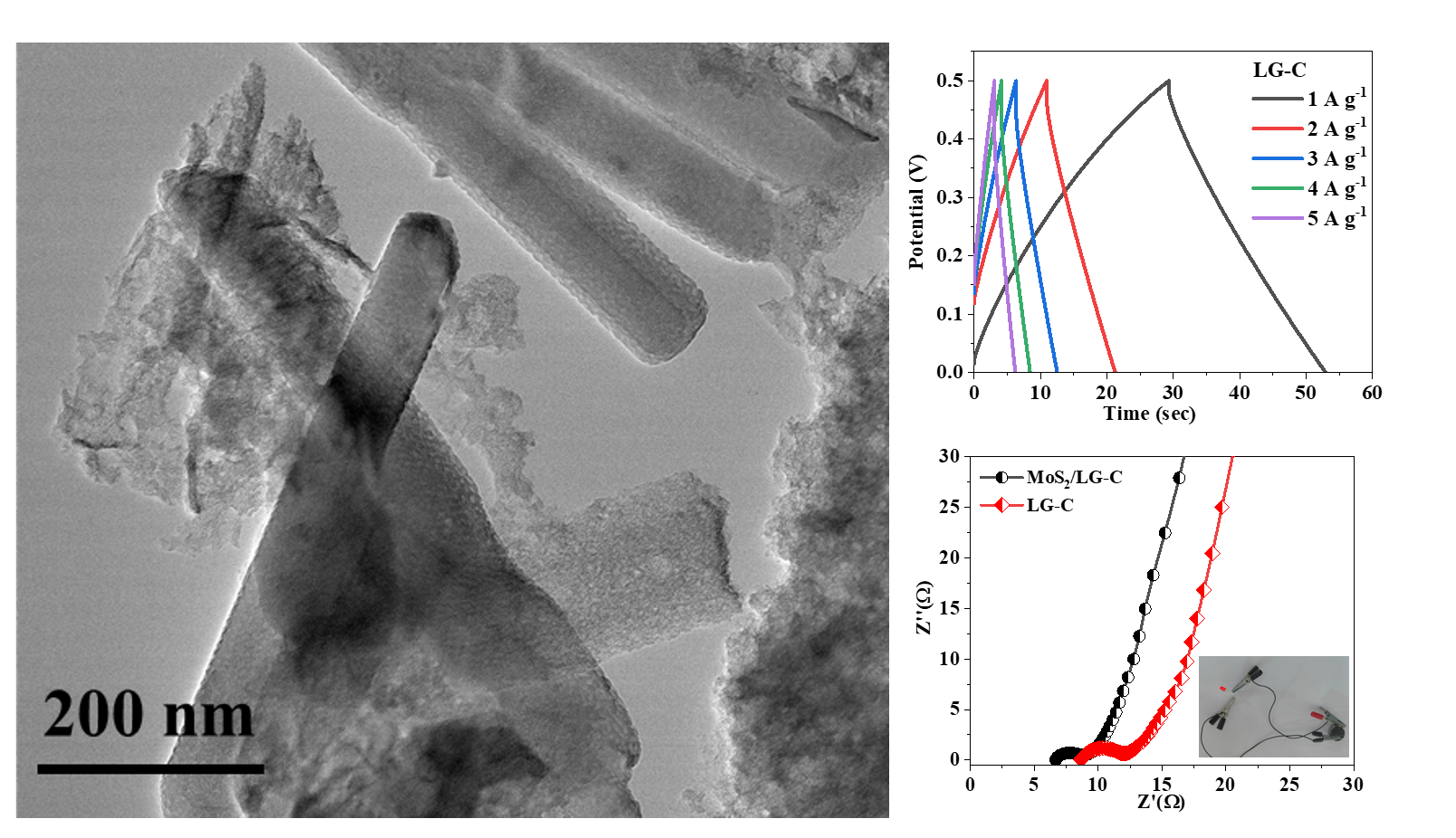

Supplement: Supplementary file 1 [file Image1.PNG]
